# Generalized convolutional many-body distribution functional representations

**DOI:** 10.1073/pnas.2415662122

**Published:** 2025-10-06

**Authors:** Danish Khan, O. Anatole von Lilienfeld

**Affiliations:** ^a^Department of Chemistry, Chemical Physics Theory Group, University of Toronto, St. George Campus, Toronto, ON M5R 0A3, Canada; ^b^Vector Institute for Artificial Intelligence, Toronto, ON M5S 1M1, Canada; ^c^Department of Materials Science and Engineering, University of Toronto, St. George Campus, Toronto, ON M5R 0A3, Canada; ^d^Institute for the Foundations of Learning and Data, Machine Learning Group, Technische Universität Berlin, Berlin 10587, Germany; ^e^Foundations of Learning and Data, Berlin Institute, Berlin 10587, Germany; ^f^Department of Physics, University of Toronto, St. George Campus, Toronto, ON M5R 0A3, Canada; ^g^Acceleration Consortium, University of Toronto, Toronto, ON M5R 0A3, Canada

**Keywords:** machine learning, quantum chemistry, chemical physics

## Abstract

Modern machine learning (ML) models of chemical and materials systems with billions of parameters require vast training datasets and considerable computational efforts. Lightweight kernel or decision tree-based methods, however, can be rapidly trained, leading to a considerably lower carbon footprint. We introduce generalized convolutional many-body distribution functionals (cMBDF) as highly compute and data-efficient atomic representations for accurate kernels that excel in low-data regimes. Generalizing the MBDF framework, cMBDF encodes local chemical environments in a compact fashion using translationally and rotationally invariant functionals of smooth atomic densities weighted by interaction potentials. The functional values can be efficiently evaluated by expressing them in terms of convolutions which are calculated via fast Fourier transforms and stored on predefined grids. In the generalized form, each atomic environment is described using a set of functionals uniformly defined by three integers; many-body, derivative, weighting orders. Irrespective of size/composition, cMBDF atomic vectors remain compact and constant in size for a fixed choice of these orders controlling the structural and compositional resolution. While being up to two orders of magnitude more compact than other popular representations, cMBDF is shown to be more accurate for the learning of various quantum properties such as energies, dipole moments, homo–lumo gaps, heat capacity, polarizability, optimal exact-exchange admixtures, and basis-set scaling factors. Applicability for organic and inorganic chemistry is tested as represented by the QM7b, QM9, and VQM24 datasets. Due to its compactness, model training and testing times are reduced from 23 h to 8 min, implying a corresponding reduction in carbon footprint.

## Significance

In this work, we propose to represent chemical environments as vectorized objects which can be used as input for machine learning (ML) properties of atomistic systems. The proposed method efficiently captures the physics of an atom’s surroundings, satisfying all symmetry requirements while remaining highly compact in size. This leads to significantly faster ML models as model training and prediction compute times are reduced by orders of magnitude. The compactness is achieved through the convolution theorem for Fourier transforms without sacrificing accuracy. The proposed methodology can reduce the energy consumption when training and applying ML models throughout chemical compound space.

While training data needs are increasingly being met by high-quality quantum mechanical (QM) datasets (1–5), it is crucial to recognize that chemical space is vast, and ML models, being dependent on the quality and diversity of their training data, can struggle to generalize across the full breadth of this space (6). This is particularly concerning as training large deep learning models already consumes significant energy which is on a clear track for becoming increasingly unsustainable (7). Carbon dioxide emissions from training large language models, for instance, are soon expected to match the monthly emissions of New York City (8). Consequently, accurate and lightweight models that require minimal training data along with significantly reduced feature or parameter counts are highly desirable across all fields.

Alternatively, they can be paired with, universally applicable, quantum chemistry methods through Δ (9), multilevel (10), and adaptive (11, 12) learning schemes which lead to significant reduction in training requirements. Substantial reductions, with or without the aforementioned schemes, are also obtained through improved atomic feature vector mappings (13, 14) (or representations) by incorporating known physical laws. Some of the most prominent examples of these are the Behler–Parrinello symmetry functions (15, 16) (ACSF), permutationally invariant polynomials (17), Coulomb matrix (18) (CM), smooth overlap of atomic positions (19) (SOAP) and atomic cluster expansion (20). While such physics-based representations can significantly reduce training data needs (21), the most recent deep learning–based methods rely on feature learning through the data itself which bloats the training requirements. The data efficiency is especially pronounced when using these “hand-crafted” representations with more efficient interpolants in the low training data regime such as kernel-based methods (22). Furthermore, the computational cost of these models is significantly affected by the choice of the molecular representation (23).

Due to the profound impact on all learning tasks and computational cost, the atomic representation choice is akin to the level-of-theory in Pople’s model quantum chemistry methods. Expectedly then, the use of physically inspired representations leads to more data-efficient ML methods which do not rely on first learning the mapping through the training data itself. (14) This is crucial due to the vast size of chemical space which can only be probed through interpolative models if they i) can be regressed with limited training data, ii) can be applied across structural and compositional degrees of freedom and iii) are computationally feasible enough (including training time) to provide significant acceleration over their, extrapolative, quantum chemistry counterparts.

Satisfying these requirements while striking the optimal trade-off between data and computational efficiency (see below), in this work, we introduce the convolutional many body distribution functionals (cMBDF) representation. Generalizing the MBDF framework (23), we use a uniform series of translationally and rotationally invariant functionals of the atomic density to efficiently quantify the local chemical environment of an atom. The original MBDF representation focused on compactness and used only five functionals, chosen empirically to maximize accuracy, to describe each atomic environment. This framework is generalized to obtain a systematically improvable family of atomic descriptors controlled by three integers (weighting function, many-body, and derivative orders) which allow controlling the computational vs. data efficiency trade-off using a set of uniformly defined functionals. For a fixed value of these indices; the atomic representation remains constant size due to its invariance to the radial cut-offs, number of neighbors and unique chemical elements. Apart from element-specific basis functions, improved weighting functions and four-body functionals, cMBDF bypasses all integral evaluations (performed numerically in MBDF) by expressing the functionals as a series of convolutions. Using the convolution theorem, these are efficiently evaluated via fast Fourier transforms and stored on predefined grids leading to significant speed-ups for on-the-fly application and gradient evaluation. Physical interactions (both short and long-range) of interest can be incorporated by raising/lowering the weighting function order.

The compactness of cMBDF feature vectors is inherent to the methodology in contrast to compression techniques (24) which can be applied to all atomic representations. Hence, it is able to outperform other commonly used representations while remaining up to 2 orders of magnitude more compact as demonstrated for several learning tasks below. This computational and data efficiency has allowed its successful application to adaptive-ML schemes (11, 12) across chemical space which improve existing quantum chemistry methods with limited, high quality, training data.

## Theory and Methods

### Two and Three-Body Functionals.

We are interested in defining a feature vector mapping $P_{i}$ of an atom i within a chemical system while minimizing the vector length. The starting point is the smooth atom-centered atomic density (21) (an electron density proxy) $\rho_{i}\left(r\right)$

[1] $$\begin{array}{c} \rho_{i}\left(r\right)=\sum_{j}^{N}A\left(Z_{j}\right)N_{r}\left(R_{\mathrm{ij}},\sigma \left(Z_{j}\right)\right), \end{array}$$

where $N_{x}\left(\mu ,\sigma \right)=\frac{1}{\sqrt{2\pi \sigma^{2}}}\exp\left(-\frac{{\left(x-\mu \right)}^{2}}{2\sigma^{2}}\right)$ denotes the normal probability distribution function (PDF) centered at μ with SD σ throughout this work, $A(Z_{j})$ is a chemical element–specific scaling (Eq. **28**), $R_{\mathrm{ij}}=R_{j}-R_{i}$ is the relative position of atom j with respect to i, and N denotes the number of atoms within a local cut-off radius $r_{\mathrm{cut}}$ around i. Gaussian basis functions are widely used to represent atomic densities in machine learning (21) and electronic densities (orbitals) in quantum chemistry (25), owing to their smoothness (infinite differentiability) and the ability to perform analytical integration using the Gaussian product theorem. These, along with their convolution property, make them highly desirable in the subsequent derivation and will be used throughout.

Incorporating rotational invariance, the density can be projected onto internal coordinates starting with the two-body distribution function $\rho_{i}\left(r\right)$

[2] $$\begin{array}{cc} \rho_{i}\left(r\right) & =\langle \rho_{i}\left(r\right)|\sum_{j}\delta \left(R_{\mathrm{ij}}-r\right)|\rho_{i}\left(r^{\prime }\right)\rangle , \end{array}$$

which can be simplified using the fact that the convolution of two Gaussians is another Gaussian

[3] $$\begin{array}{cc} \rho_{i}\left(r\right) & \approx \sum_{j}^{N}A_{2}\left(Z_{j}\right)\;N_{r}\left(R_{\mathrm{ij}},\sigma \right), \end{array}$$

where $R_{\mathrm{ij}}=\left|R_{j}-R_{i}\right|$ is the interatomic distance between atoms i and j. Translationally and rotationally invariant feature vector components encoding two-body features of the atomic environment can then be defined through functionals of the general form

[4] $$\begin{array}{c} P_{2}^{\mathrm{nm}}\left[i\right]=\int_{0}^{\infty }dr\;g_{n2}\left(r\right)\;\partial_{r}^{m}\rho_{i}\left(r\right), \end{array}$$

where $g_{n2}\left(r\right)$ are suitable weighting functions (Eq. **26**). Inclusion of the derivatives $\partial_{r}^{m}\rho_{i}\left(r\right)$ allows the unique description of the *total* $\rho_{i}\left(r\right)$ in Eq. **3** without generating separate distributions for each chemical element in the neighborhood. Eq. **4** can be expressed in terms of m-th degree Hermite polynomials $H_{m}$ centered at $R_{\mathrm{ij}}$ (due to the relation $\partial_{r}^{m}\exp\left(-{\left(r-a\right)}^{2}\right)={\left(-1\right)}^{m}\exp\left(-{\left(r-a\right)}^{2}\right)H_{m}\left(r-a\right)$ (26))

[5] $$\begin{array}{cc} P_{2}^{\mathrm{nm}}\left[i\right] & ={\left(\frac{-1}{\sqrt{2}\sigma }\right)}^{m}\int_{0}^{\infty }dr\;g_{n2}\left(r\right)\sum_{j}^{N}A_{2}\left(Z_{j}\right)f_{m}\left(r-R_{\mathrm{ij}}\right), \end{array}$$

[6] $$\begin{array}{cc} & ={\left(\frac{-1}{\sqrt{2}\sigma }\right)}^{m}\sum_{j}^{N}A_{2}\left(Z_{j}\right)\int_{0}^{\infty }dr\;g_{n2}\left(r\right)f_{m}\left(r-R_{\mathrm{ij}}\right), \end{array}$$

where we have used the following relation (see *SI Appendix*, *Hermite Polynomials* for details)

[7] $$\begin{array}{cc} \partial_{r}^{m}\rho_{i}\left(r\right) & =\sum_{j}^{N}A_{2}\left(Z_{j}\right)\;\partial_{r}^{m}N_{r}\left(R_{\mathrm{ij}},\sigma \right)\\ & ={\left(\frac{-1}{\sqrt{2}\sigma }\right)}^{m}\sum_{j}^{N}A_{2}\left(Z_{j}\right)N_{r}\left(R_{\mathrm{ij}},\sigma \right)H_{m}\left(\frac{r-R_{\mathrm{ij}}}{\sqrt{2}\sigma }\right) \end{array}$$

and $f_{m}\left(r\right)$ denotes the Hermite–Gaussian function

[8] $$\begin{array}{cc} f_{m}\left(r\right) & =N_{r}\left(0,\sigma \right)H_{m}\left(\frac{r}{\sqrt{2}\sigma }\right)\\ \Rightarrow f_{m}\left(r-R_{\mathrm{ij}}\right) & =N_{r}\left(R_{\mathrm{ij}},\sigma \right)H_{m}\left(\frac{r-R_{\mathrm{ij}}}{\sqrt{2}\sigma }\right). \end{array}$$

Now, using $f_{m}\left(-u\right)={\left(-1\right)}^{m}f_{m}\left(u\right)$ [due to $H_{m}\left(-u\right)={\left(-1\right)}^{m}H_{m}\left(u\right)$ (26)] in Eq. **6**, we have

[9] $$\begin{array}{c} P_{2}^{\mathrm{nm}}\left[i\right]=\sum_{j}^{N}\frac{A_{2}\left(Z_{j}\right)}{{\left(\sqrt{2}\sigma \right)}^{m}}\int_{0}^{\infty }dr\;g_{n2}\left(r\right)f_{m}\left(R_{\mathrm{ij}}-r\right), \end{array}$$

which is a sum of convolutions

[10] $$\begin{array}{cc} P_{2}^{\mathrm{nm}}\left[i\right] & =\sum_{j}^{N}\;\frac{A_{2}\left(Z_{j}\right)}{{\left(\sqrt{2}\sigma \right)}^{m}}\;\left(g_{n2}*f_{m}\right)\left(R_{\mathrm{ij}}\right) \end{array}$$

[11] $$\begin{array}{cc} P_{2}^{\mathrm{nm}}\left[i\right] & =\sum_{j}^{N}\;\frac{A_{2}\left(Z_{j}\right)}{{\left(\sqrt{2}\sigma \right)}^{m}}\;H_{2}^{\mathrm{nm}}\left(R_{\mathrm{ij}}\right) \end{array}$$

and the convolved function $H_{2}^{\mathrm{nm}}$ can be calculated via application of the convolution theorem assuming $g_{n2}\left(r\right)$ is square integrable

[12] $$\begin{array}{c} H_{2}^{\mathrm{nm}}\left(R_{\mathrm{ij}}\right)=F^{-1}\left\{F\left\{g_{n2}\right\}F\left\{f_{m}\right\}\right\}\left(R_{\mathrm{ij}}\right), \end{array}$$

where F denotes a Fourier transform. Note that the function $H_{2}^{\mathrm{nm}}$ is unique and independent of the system. Therefore, it needs to be evaluated only once on a predefined grid and stored, subsequently allowing all integral evaluations to be bypassed.

In a similar fashion, three-body functionals can be defined using the three-body distribution function $\rho_{i}\left(\theta \right)$

[13] $$\begin{array}{cc} \rho_{i}\left(\theta \right) & =\langle \rho_{i}\left(r\right)|\sum_{\mathrm{jk}}f\left(R_{\mathrm{ij}}\right)f\left(R_{\mathrm{ik}}\right)f\left(R_{\mathrm{jk}}\right)\delta \left(\theta -\theta_{\mathrm{ijk}}\right)|\rho_{i}\left(r^{\prime }\right)\rangle , \end{array}$$

[14] $$\begin{array}{cc} \rho_{i}\left(\theta \right) & \approx \sum_{\mathrm{jk}}^{N}A_{3}\left(Z_{j},Z_{k}\right)f\left(R_{\mathrm{ij}}\right)f\left(R_{\mathrm{ik}}\right)f\left(R_{\mathrm{jk}}\right)N_{\theta }\left(\theta_{\mathrm{ijk}},\sigma \right), \end{array}$$

where, again, we have used the fact that the convolution of two Gaussians is another Gaussian, $\theta_{\mathrm{ijk}}=\;\cos^{-1}\left(\frac{R_{\mathrm{ij}}\cdot R_{\mathrm{ik}}}{|R_{\mathrm{ij}}\left|\right|R_{\mathrm{ik}}|}\right)$ is the three-body interatomic angle centered at atom i and $f\left(R_{\mathrm{ij}}\right)=1/R_{\mathrm{ij}}^{2}$ is chosen such that the Axilrod–Teller–Muto (27) (ATM) scaling is recovered through the product $f\left(R_{\mathrm{ij}}\right)f\left(R_{\mathrm{ik}}\right)f\left(R_{\mathrm{jk}}\right)$. This scaling has been successfully applied in other works (28, 29).

Subsequently, translationally and rotationally invariant functionals encoding three-body interactions, through suitable weighting functions $g_{n3}\left(\theta \right)$ (Eq. **27**), can be defined in the general form similar to Eq. **4**

[15] $$\begin{array}{c} P_{3}^{\mathrm{nm}}\left[i\right]=\int_{0}^{\pi }d\theta \;g_{n3}\left(\theta \right)\;\partial_{\theta }^{m}\rho_{i}\left(\theta \right). \end{array}$$

Functionals of the derivatives $\partial_{\theta }^{m}\rho_{i}\left(\theta \right)$ again allow the unique description of the total distribution $\rho_{i}\left(\theta \right)$ without generating separate distributions for each unique triplet of chemical elements. This is common practice with all other ν-body atomic representations which induces a feature vector size scaling of $N_{\mathrm{elem}}^{\nu -1}$ for systems containing $N_{\mathrm{elem}}$ number of unique chemical elements.

Using the same procedure as above for $P_{2}^{\mathrm{nm}}\left[i\right]$ we arrive at

[16] $$\begin{array}{c} P_{3}^{\mathrm{nm}}\left[i\right]=\sum_{\mathrm{jk}}^{N}\frac{A_{3}\left(Z_{j},\;Z_{k}\right)}{{\left(\sqrt{2}\sigma \right)}^{m}{\left(R_{\mathrm{ij}}R_{\mathrm{ik}}R_{\mathrm{jk}}\right)}^{2}}\int_{0}^{\pi }d\theta g_{n3}\left(\theta \right)f_{m}\left(\theta_{\mathrm{ijk}}-\theta \right), \end{array}$$

where $f_{m}\left(\theta \right)=N_{\theta }\left(0,\sigma \right)H_{m}\left(\frac{\theta }{\sqrt{2}\sigma }\right)$ denotes the three-body Hermite–Gaussian function. Hence, we again recover a sum of convolutions

[17] $$\begin{array}{c} P_{3}^{\mathrm{nm}}\left[i\right]=\sum_{\mathrm{jk}}^{N}\frac{A_{3}\left(Z_{j},\;Z_{k}\right)}{{\left(R_{\mathrm{ij}}R_{\mathrm{ik}}R_{\mathrm{jk}}\right)}^{2}}\;\frac{H_{3}^{\mathrm{nm}}\left(\theta_{\mathrm{ijk}}\right)}{{\left(\sqrt{2}\sigma \right)}^{m}}, \end{array}$$

where, similar to Eq. **12**, the function

[18] $$\begin{array}{c} H_{3}^{\mathrm{nm}}\left(\theta_{\mathrm{ijk}}\right)=\left(g_{n3}⊛f_{m}\right)\left(\theta_{\mathrm{ijk}}\right)=F^{-1}\left\{F\left\{g_{n3}\right\}F\left\{f_{m}\right\}\right\}\left(\theta_{\mathrm{ijk}}\right) \end{array}$$

is the circular convolution of the three-body weighting function $g_{n3}\left(\theta \right)$ and $f_{m}\left(\theta \right)$.

### Pseudo Four-Body Functionals.

Higher-order many-body functionals, desirable to account for dihedrals, for example, can be defined in a similar fashion. For the four-body terms, we define a pseudo four-body distribution using radial distribution functions for computational efficiency

[19] $$\begin{array}{c} \rho_{i4}\left(r\right)\approx \sum_{\mathrm{jkl}}^{N}A_{4}\left(Z_{j},Z_{k},Z_{l}\right)\prod_{\left\{a,b\right\}\in S_{\mathrm{ijkl}}}f_{\mathrm{ab}}N_{r}\left(R_{\mathrm{ab}},\sigma \right) \end{array}$$

with $f_{\mathrm{ab}}=\frac{1}{R_{\mathrm{ab}}^{2}}$ similar to the three-body term and $S_{\mathrm{ijkl}}=\left\{a,b\right\}\in \left(\begin{array}{c} \left\{i,j,k,l\right\}\\ 2 \end{array}\right)$ is the set of all six unique interatomic distances between the four atoms i,j,k,l. Eq. **19** can be simplified into a form similar to the two-body distribution in Eq. **3** as

[20] $$\begin{array}{c} \rho_{i4}\left(r\right)=\sum_{\mathrm{jkl}}^{N}A_{4}\left(Z_{j},Z_{k},Z_{l}\right)f_{\mathrm{ijkl}}N_{r}\left(R_{\mathrm{ijkl}},\sigma_{\mathrm{ijkl}}\right) \end{array}$$

where $f_{\mathrm{ijkl}}=\prod_{\left\{a,b\right\}\in S_{\mathrm{ijkl}}}\frac{1}{R_{\mathrm{ab}}^{2}}$ is the four-body scaling and the SD of these distributions corresponds to $\sigma_{\mathrm{ijkl}}$ due to Eq. **21**.

The effective four-body distribution $N_{r}\left(R_{\mathrm{ijkl}},\sigma_{\mathrm{ijkl}}\right)=\prod_{\left\{a,b\right\}\in S_{\mathrm{ijkl}}}N_{r}\left(R_{\mathrm{ab}},\sigma \left(Z_{b}\right)\right)$ is obtained via successive application of the Gaussian product theorem

[21] $$\begin{array}{cc} & \exp\left(-\alpha |r-R_{a}{|}^{2}\right)\exp\left(-\beta |r-R_{b}{|}^{2}\right)\\ & =\zeta \exp\left(-\left(\alpha +\beta \right)|r-R_{p}{|}^{2}\right), \end{array}$$

where

[22] $$\begin{array}{cc} \zeta & =\;\exp\left(-\frac{\alpha \beta }{\alpha +\beta }{\left|R_{a}-R_{b}\right|}^{2}\right), \end{array}$$

[23] $$\begin{array}{cc} R_{p} & =\frac{\alpha R_{a}+\beta R_{B}}{\alpha +\beta }. \end{array}$$

Now using the distribution in Eq. **20**, the four-body functionals are calculated in a similar way to Eq. **4**

[24] $$\begin{array}{cc} P_{4}^{\mathrm{nm}}\left[i\right] & =\int_{0}^{\infty }dr\;g_{n4}\left(r\right)\;\partial_{r}^{m}\rho_{i4}\left(r\right)\\ & =\sum_{\mathrm{jkl}}^{N}\frac{A_{4}\left(Z_{j},\;Z_{k},\;Z_{l}\right)f_{\mathrm{ijkl}}}{{\left(\sqrt{2}\sigma_{\mathrm{ijkl}}\right)}^{m}}\;H_{4}^{\mathrm{nm}}\left(R_{\mathrm{ijkl}}\right) \end{array}$$

with

[25] $$\begin{array}{c} H_{\nu }^{\mathrm{nm}}\left(t\right)=F^{-1}\left\{F\left\{g_{n\nu }\right\}F\left\{f_{m}\right\}\right\}\left(t\right) \end{array}$$

as before with $f_{m}\left(r\right)=N_{r}\left(0,\sigma_{\mathrm{ijkl}}\right)H_{m}\left(\frac{r}{\sqrt{2}\sigma }\right)$. Higher-order pseudo ν-body functionals can be defined through a similar procedure using radial distributions.

### Weighting Functions and Scaling Factors.

Within the presented methodology, the choice of the ν-body weighting functions $g_{n\nu }$ is the only hyperparameter. We have used very simple weighting functions $g_{n2}\left(r\right),g_{n3}\left(\theta \right),g_{n4}\left(r\right)$ in our work parameterized by an integer n. Two separate types of weighting functions are used for each ν-body case. For the two- and four-body (ν=2,4, respectively) functionals, we use simple decaying functions (square-integrable over the positive reals) of the form

[26] $$\begin{array}{c} g_{n\nu }\left(r\right)=\left\{\begin{array}{c} \exp(-\alpha_{\nu }\left(n+1\right)r)\\ \frac{1}{{\left(r+1\right)}^{\left(2n+3\right)}} \end{array}\right., \end{array}$$

where $\alpha_{\nu }=\alpha_{2}$, $\alpha_{4}$ are hyperparameters (set to 1.5 as obtained in ref. 23) of the representation for the two- and four-body functionals. For the three-body functions, we use angular Fourier series terms employed in ACSF-based representations (16, 30)

[27] $$\begin{array}{c} g_{n3}\left(\theta \right)=\left\{\begin{array}{c} \cos((2n+1)\theta )-\cos((2n+1)(\theta +\pi ))\\ \sin((2n+1)\theta )-\sin((2n+1)(\theta +\pi )) \end{array}\right.\;. \end{array}$$

**Fig. 1.**
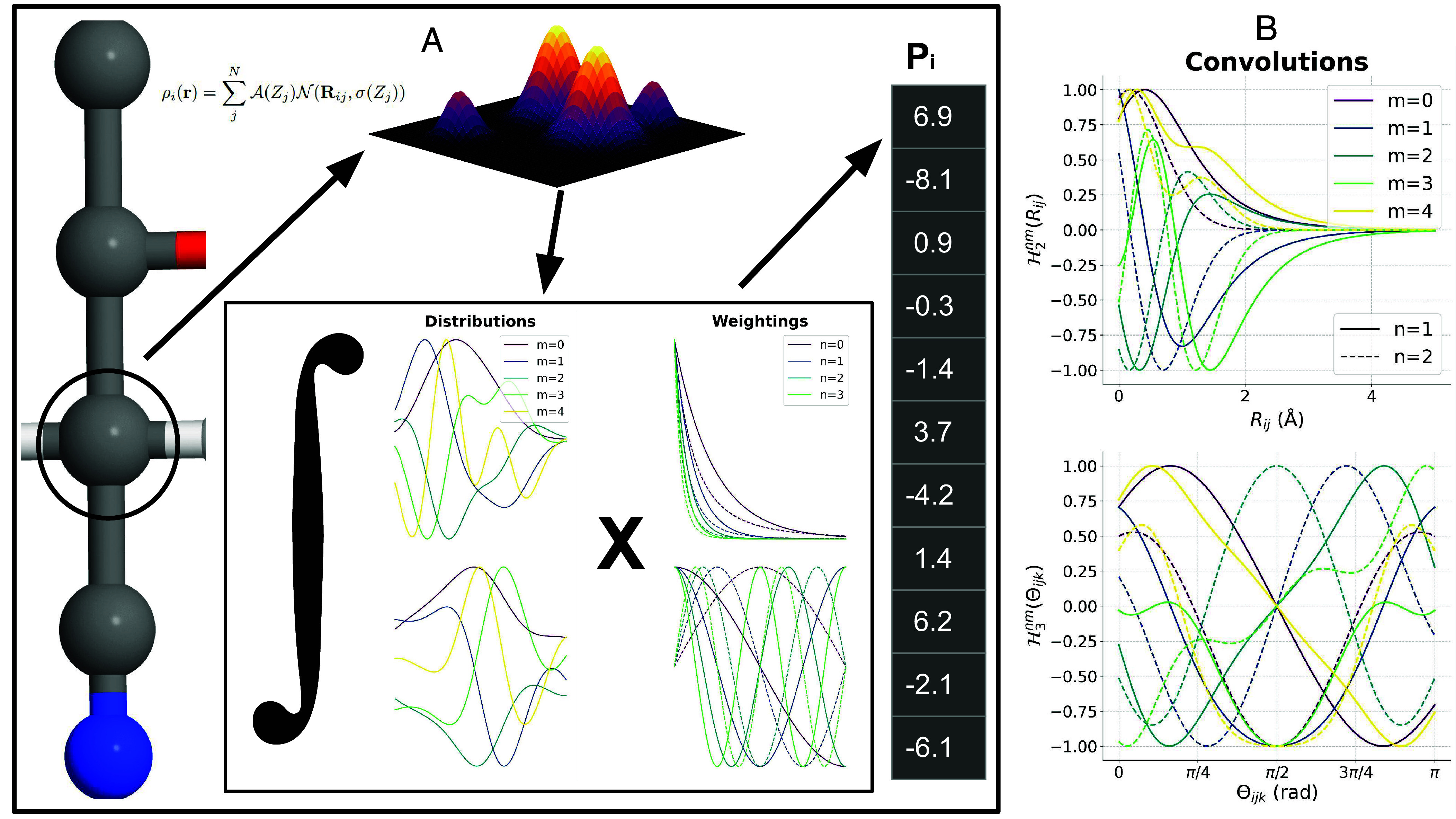
(*A*) Schematic representation of convolutional many-body distribution functionals (cMBDF) atomic feature vector generation for an atom i within any system. A smooth atomic density $\rho_{i}\left(r\right)$ (Eq. **1**), centered on atom i, is constructed by placing element-specific basis functions on all atoms within its local neighborhood. Translationally and rotationally invariant distributions based on this atomic density are generated by projecting it onto many-body internal coordinates (Eqs. **3**, **14**, and **19**). The distributions and their derivatives are weighted by many-body interaction potentials of interest (Eqs. **26** and **27**). The chemical environment of the atomic species is then encoded in a feature vector consisting of these functional values integrated over the domain of the distributions (Eqs. **4**, **17**, and **24**). The integral evaluations can be bypassed for “on-the-fly” generation of the feature vectors by expressing them as sums of convolutions (Eqs. **12**, **18**, **25**). Panel (*B*) shows the two (*Left*) and three-body (*Right*) convolved functions $H_{\nu }^{\mathrm{nm}}$ for weighting functions of the first type in Eqs. **26** and **27**.

Fig. 1*B* shows the convolved functions $H_{\nu }^{\mathrm{nm}}$ for the n=1,2 cases with the weighting functions of the first type from Eqs. **26** and **27**. We point out here again that once the weighting functions are chosen, these convolutions can be preevaluated on a discretized grid as shown in Fig. 1*B*. For generating the representation vector of an atom within a molecule, only the molecular internal coordinates are required to be calculated. The functional values $P_{\nu }^{\mathrm{nm}}$ can then be obtained by indexing the internal coordinate values on the precomputed $H_{\nu }^{\mathrm{nm}}$ grid followed by summation. The speedup obtained from this is shown in Table 1 which tabulates the representation generation timings for organic molecules taken from four datasets with different sizes. These timings are compared to the original MBDF representation which constituted a nonuniform subset of cMBDF in which the functional values were calculated via numerical integration. In all four cases, cMBDF is significantly faster despite evaluating 7× (40 for cMBDF vs. 5 for MBDF) more functionals per atom. Table 1 also tabulates timings for the SLATM (28), FCHL19 (30), and SOAP (19) representations. Evidently, cMBDF is fastest to generate for molecules from the QM9 (1), QM7b (4), and VQM24 (5) datasets out of all five representations. Furthermore, our cMBDF code is currently implemented entirely in Python, whereas SLATM, FCHL19, and SOAP are generated using Python libraries that leverage lower-level programming languages (Fortran, C) for the computations and looping (see *Data and Code* for details). Hence, further optimizations are possible.

**Table 1.** Representation generation timing (in node seconds) comparison between cMBDF and five other representations [MBDF (23), SLATM (28), FCHL19 (30), SOAP (19)] for molecules from four different datasets [QM9 (1), QM7b (4), VQM24 (5)] containing organic and inorganic molecules

Dataset (size)	cMBDF	MBDF	SLATM	FCHL19	SOAP
QM9 (130k)	7.3	102.8	17.6	24.7	7.7
QM7b (7.2k)	0.5	3.4	1.3	1.1	0.6
VQM24 (258k)	6.7	26.5	273.1	83.4	16.4

Numbers in brackets of column 1 denote the number of molecules used from the dataset with k denoting thousand. All representations were generated through embarrassing parallelization over all molecules from the dataset using the JoblibPython library. All timings were evaluated on a compute node equipped with an AMD Ryzen 9 7950X 16-Core CPU and 128 GB DDR5 RAM.

The scaling prefactors $A_{\nu }(Z_{1},Z_{2},$...$,Z_{\nu -1})$ used alongside all distributions (Eqs. **3**, **14**, and **20**) are calculated as geometric mean of a function encoding the chemical identity of the elements

[28] $$\begin{array}{cc} A(Z) & =\;\log(P+1)G, \end{array}$$

[29] $$\begin{array}{cc} A_{\nu }\left(Z_{1},Z_{2}..Z_{\nu -1}\right) & ={\left(\prod_{j=1}^{\nu -1}A\left(Z_{j}\right)\right)}^{\frac{1}{\nu -1}}, \end{array}$$

where P, G denote the period and group number of the chemical element Z in the periodic table. The form chosen in Eq. **28** ensures scaling factors for elements from the same group are more similar than from the same period.

The functional values evaluated from Eqs. **11**, **17**, and **24** are then concatenated to form the feature vector $P_{i}$ describing atom i

[30] $$\begin{array}{c} P_{i}=\left[P_{2}^{00}\left[i\right]...P_{2}^{\mathrm{nm}}\left[i\right],P_{3}^{00}\left[i\right]...P_{3}^{\mathrm{nm}}\left[i\right],P_{4}^{00}\left[i\right]....P_{4}^{\mathrm{nm}}\left[i\right]\right]. \end{array}$$

The entire procedure is summarized as a schematic in Fig. 1*A*.

This leads to a class of compact and systematically improvable atomic descriptors as controlled by the three integers ν (many body-order), m (derivative-order), and n (weighting function-order). The size (dimensionality) of the atomic feature vector is given by the product 2(ν−1)(m+1)n ($P_{i}\;\epsilon \;R^{2(\nu -1)(m+1)n}$) and is invariant to the chemical species, system size, and cut-offs employed.

Throughout the rest of the work, we have used m=4, n=2 along with ν=3 (denoted cMBDF) or ν=4 [denoted cMBDF (four-body)] leading to atomic feature vector lengths of 40 and 60, respectively.

### Gradients for Responses.

Gradients required for calculating response properties can be evaluated efficiently as well. We first note that the gradients of the $H_{\nu }^{\mathrm{nm}}$ convolved functions with respect to the nuclear position $R_{a}$ can be evaluated easily via application of the chain rule

[31] $$\begin{array}{c} \nabla_{R_{a}}H_{\nu }^{\mathrm{nm}}\left(x\left(R_{a}\right)\right)=\partial_{x}H_{\nu }^{\mathrm{nm}}\left(x\right)\nabla_{R_{a}}x\left(R_{a}\right) \end{array}$$

and

$$\begin{array}{c} \partial_{x}H_{\nu }^{\mathrm{nm}}\left(x\right)=F^{-1}\left\{F\left\{g_{n\nu }\right\}F\left\{\partial_{x}f_{m}\right\}\right\}\left(x\right)=\frac{-1}{\sqrt{2}\sigma }H_{\nu }^{n(m+1)}\left(x\right), \end{array}$$

where the first equality follows from Leibniz rule and we have used the relation $\partial_{x}f_{m}=\frac{-1}{\sqrt{2}\sigma }f_{m+1}$ (*SI Appendix*, *Hermite Polynomials*) for the second equality. Hence, the derivative on $H_{\nu }^{\mathrm{nm}}$ essentially raises the Hermite polynomial degree by 1. Consequently,

[32] $$\begin{array}{c} \nabla_{R_{a}}H_{\nu }^{\mathrm{nm}}\left(x\left(R_{a}\right)\right)=\frac{-1}{\sqrt{2}\sigma }H_{\nu }^{n(m+1)}\left(x\right)\nabla_{R_{a}}x\left(R_{a}\right). \end{array}$$

Note that due to the symmetry of convolutions, the following expression also holds

[33] $$\begin{array}{c} \frac{\partial H_{\nu }^{\mathrm{nm}}\left(x\right)}{\partial x}=F^{-1}\left\{F\left\{\partial_{x}g_{n\nu }\right\}F\left\{f_{m}\right\}\right\}\left(x\right), \end{array}$$

however a similar relation to $\partial_{x}f_{m}=\frac{-1}{\sqrt{2}\sigma }f_{m+1}$ does not hold for the weighting function derivatives $\partial_{x}g_{n\nu }$ in general and is not used in our work.

Using Eq. **32**, the gradients of the functionals $P_{\nu }^{\mathrm{nm}}$ can be readily evaluated as

[34] $$\begin{array}{cc} \nabla_{R_{a}}P_{2}^{\mathrm{nm}} & =\sum_{j}^{N}\;\frac{-A_{2}\left(Z_{j}\right)}{{\left(\sqrt{2}\sigma \right)}^{m}}\;H_{2}^{n(m+1)}\left(R_{\mathrm{ij}}\right)\nabla_{R_{a}}R_{\mathrm{ij}}, \end{array}$$

[35] $$\begin{array}{cc} \nabla_{R_{a}}P_{3}^{\mathrm{nm}} & =\sum_{\mathrm{jk}}^{N}\;\frac{-A_{3}\left(Z_{j},\;Z_{k}\right)}{{\left(\sqrt{2}\sigma \right)}^{m}}[H_{3}^{\mathrm{nm}}\left(\theta_{\mathrm{ijk}}\right)\nabla_{R_{a}}f_{\mathrm{ijk}}\\ & -f_{\mathrm{ijk}}H_{3}^{n(m+1)}\left(\theta_{\mathrm{ijk}}\right)\nabla_{R_{a}}\theta_{\mathrm{ijk}}], \end{array}$$

[36] $$\begin{array}{cc} \nabla_{R_{a}}P_{4}^{\mathrm{nm}} & =\sum_{\mathrm{jkl}}^{N}\;\frac{-A_{4}\left(Z_{j},\;Z_{k},\;Z_{l}\right)}{{\left(\sqrt{2}\sigma \right)}^{m}}[H_{4}^{\mathrm{nm}}\left(R_{\mathrm{ijkl}}\right)\nabla_{R_{a}}f_{\mathrm{ijkl}}\\ & -f_{\mathrm{ijkl}}H_{4}^{n(m+1)}\left(R_{\mathrm{ijkl}}\right)\nabla_{R_{a}}R_{\mathrm{ijkl}}], \end{array}$$

where $\nabla_{R_{a}}\;f_{\mathrm{ijk}}=\nabla_{R_{a}}\frac{1}{{\left(R_{\mathrm{ij}}R_{\mathrm{ik}}R_{\mathrm{jk}}\right)}^{2}}$ and $\nabla_{R_{a}}f_{\mathrm{ijkl}}=\nabla_{R_{a}}\prod_{\left\{a,b\right\}\in S_{\mathrm{ijkl}}}\frac{1}{R_{\mathrm{ab}}^{2}}$ are straightforward to evaluate using the internal coordinate gradients $\nabla_{R_{a}}R_{\mathrm{ij}}$ and $\nabla_{R_{a}}\theta_{\mathrm{ijk}}$

[37] $$\begin{array}{cc} \nabla_{R_{a}}R_{\mathrm{ij}} & =\left(\delta_{\mathrm{aj}}-\delta_{\mathrm{ai}}\right)\frac{R_{j}-R_{i}}{R_{\mathrm{ij}}}, \end{array}$$

[38] $$\begin{array}{cc} \nabla_{R_{a}}\theta_{\mathrm{ijk}} & =\frac{\cos\;\theta_{\mathrm{ijk}}\nabla_{R_{a}}R_{\mathrm{ij}}}{R_{\mathrm{ij}}\left|\sin\;\theta_{\mathrm{ijk}}\right|}-\frac{\nabla_{R_{a}}R_{\mathrm{ik}}}{|\sin\;\theta_{\mathrm{ijk}}|R_{\mathrm{ij}}}. \end{array}$$

## Results

### Data Efficiency.

**Fig. 2.**
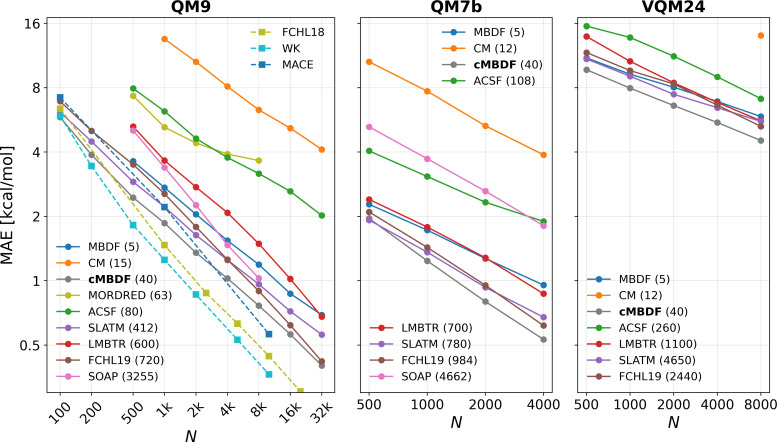
Kernel ridge regression (KRR)–based learning curves (model prediction error as a function of training set size) for molecular atomization energies from the QM9 (1), QM7b (4, 31), and VQM24 (5) datasets of small organic and inorganic molecules. Comparison to some commonly used representations alongside KRR models is shown across all three datasets. Numbers in legend denote size (dimensionality) of the atomic feature vector mapping induced by the corresponding representation. For the molecular representations (CM, SLATM, MORDRED), the dimensionality noted is the molecular feature vector size divided by the number of atoms. Representation names in legends are ordered by the feature vector lengths. Dotted lines indicate learning curves obtained from literature (14, 29, 32).

**Fig. 3.**
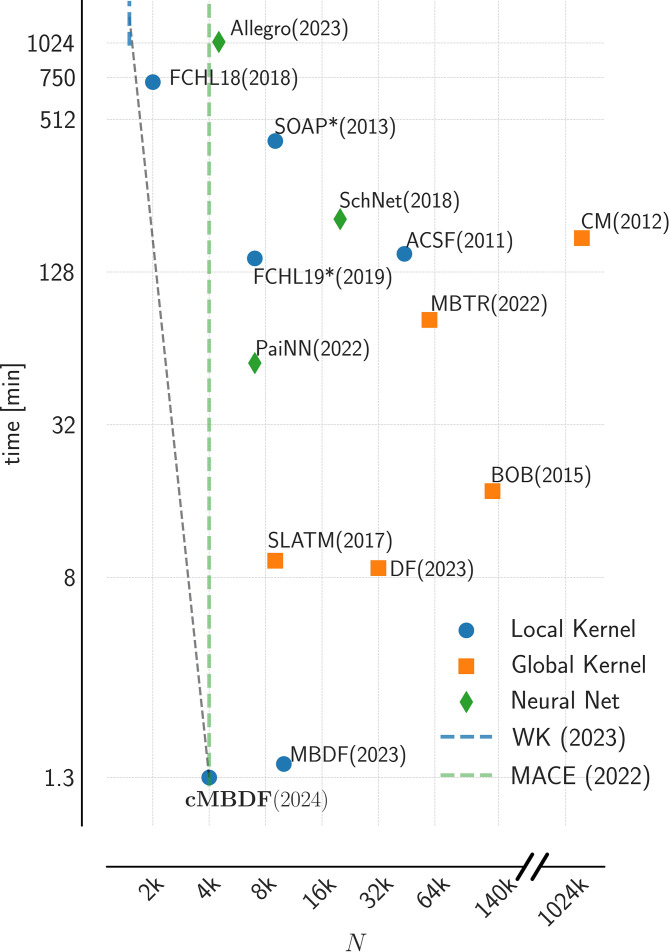
Plot showing trade-off between computational and data efficiency for various ML methods. The x-axis denotes the (minimum) number of training samples required to achieve chemically accurate (MAE = 1 kcal/mol) atomization energy predictions on the QM9 dataset. Y-axis plots the model training and prediction timing for the same task. The dashed gray line corresponds to the optimal Pareto front. Data for models other than cMBDF, Wigner Kernels (WK) (14), and MACE (33) are taken from ref. 23. The WK and MACE lines denote the (minimum) number of training samples required for chemically accurate predictions on the QM9 dataset taken from refs. 14 and 32, respectively. Exact timings for these models are unavailable. The x-axis values for BOB (34) and CM (18) were obtained through extrapolation. See *SI Appendix*, *Kernel Based Methods* and ref. 23 for further details regarding generation of this figure.

**Fig. 4.**
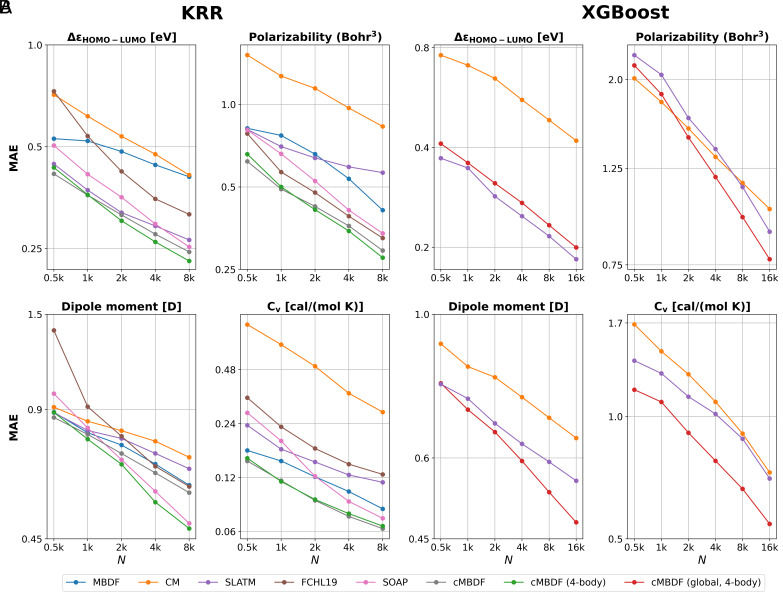
Learning curves for some intensive molecular properties from the QM9 (1) dataset using (*A*) KRR and (*B*) XGBoost (35) ML models for regression. Both local (atomic) and global (molecular) representations can be employed with the local/global variants of KRR, respectively. For XGBoost, only global representations are used. See *SI Appendix*, *Kernel Based Methods* and *XGBoost* for details regarding the ML models. Data points for KRR learning curves other than cMBDF and cMBDF (four-body) are taken from ref. 23.

We begin our discussion with the classic benchmark of learning atomization energies from a few datasets of small organic and inorganic molecules. For computational details regarding the ML models and representations, the reader is referred to *Data and Code* and *SI Appendix*, *Kernel Based Methods*. Fig. 2 shows learning curves (ML model prediction error as a function of training set size) of atomization energies from the QM9 (1), QM7b (4) and VQM24 (5) datasets. For comparison, we also plot some of the most commonly employed atomic [atom-centered symmetry functions (ACSF) (16), local many-body tensor representation (LMBTR) (36), Faber–Christensen–Huang-Lilienfeld 2019 variant (FCHL19) (30), smooth overlap of atomic positions (SOAP) (19)], molecular [Coulomb matrix (CM) (18), spectrum of London and Axilrod–Teller–Muto (SLATM) (28)] and graph-based [MORDRED (37)] representations alongside kernel based ML models. We restrict our analysis to up to ∼10,000 training data points (except on QM9) which constitutes the low-training data regime where kernel methods are more efficient than their deep-learning counterparts (22, 38). Fig. 2 also shows the size (dimensionality) of the feature vector mapping (per atom) induced by each representation in the legend. Across all three datasets, a similar ordering in terms of both the accuracy and representation size is seen for all of the tested representations. For representations other than cMBDF, the accuracy is well correlated with the representation size which, however, has a strong impact on the computational cost of each method (Fig. 3). cMBDF stands out as an exception as it remains among the most accurate (data efficient) while retaining compactness (computational efficiency). While being comparable in size to the CM representation across all three datasets, cMBDF requires up to 120× less training data to reach the same accuracies. Furthermore, while the atomic feature vector size scales with either the number of atoms or unique chemical species for all other representations, cMBDF remains constant size at 40 dimensions. The effect of this is especially pronounced on the more chemically diverse VQM24 (5) dataset which contains 10 unique chemical elements. As can be seen from Fig. 2, cMBDF remains nearly two orders of magnitude more compact than the other best-performing representations while showing a lower predictive error in the low training data regime. Exceptions to this are FCHL18 (29) and Wigner Kernels (14) which are the most accurate models on the QM9 dataset. This, however, comes at significant added computational burden as discussed in the next subsection. Furthermore, while the FCHL18 and WK representations require specific kernel forms to be applicable, cMBDF feature vectors can be employed with any ML model. This is demonstrated in Fig. 4 where cMBDF based feature vectors are paired with an XGBoost regressor (35) for prediction of a few intensive molecular properties. Meanwhile, comparison of cMBDF with the ACSF representation in Fig. 2 suggests cMBDF-based KRR models require up to 32× less training data to reach the same accuracy while still being faster due to cMBDF being smaller in size. Given these advantages, cMBDF could also find utility in widely adopted Behler–Parinello-type neural networks (15), such as ANI (2, 39), where ACSFs are routinely employed.

### Compute Efficiency.

While the FCHL18 and WK models achieve higher accuracy than cMBDF on the QM9 dataset, this improvement comes at a significant computational cost. For example, the high compute demands of kernel methods with the FCHL18 representation are the main reason it is rarely used in practice, leading to the development of the more computationally efficient FCHL19 variant (30). The introduction of FCHL19 reduced kernel matrix generation times by an order of magnitude (30).

Nevertheless, cMBDF is far more efficient than even the FCHL19 variant. For instance, on the VQM24 dataset, generating the entire learning curves (including training, optimization, and predictions) in Fig. 2 took 23 h with FCHL19 but only 8 min with cMBDF (compute node: 36-core 4.8 GHz Intel Xeon W9-3475X and 1 TB DDR5 ECC RAM). This remarkable efficiency arises from the compactness of the cMBDF feature vectors, which are 61× smaller than those of FCHL19 on this dataset.

The compute efficiency is further demonstrated in Fig. 3 which shows the model training and prediction time vs. training data requirement to reach chemically accuracy (MAE < 1 kcal/mol) on the entire QM9 (1) dataset. The dashed gray line indicates the optimal Pareto front for computational vs. data efficiency tradeoff. cMBDF significantly improves upon other representations when employed alongside kernel-based methods as it shows the fastest model timings while being the third-most data-efficient method. It reaches chemical accuracy on the entire QM9 dataset after training on only 4,000 molecules and required 1.3 min (for training and subsequent prediction on 100k QM9 molecules) of compute time. In comparison to the more accurate FCHL18 and WK methods, cMBDF is faster by factors of ∼550 and >1,000, respectively.

### Other Quantum Properties.

This computational efficiency makes cMBDF ideal for the training and testing of new models across various regions of chemical space, as well as for various tasks. While extensive properties such as atomization energies are generally easier to regress using atomic ML models such as local KRR, intensive molecular properties can be much more challenging (23, 38). Learning capacity for properties other than energetics are demonstrated in Fig. 4. Evidently, cMBDF also retains its good data efficiency for other physical properties of interest. Furthermore, the performance seems transferable to intensive properties such as HOMO–LUMO gaps and electrostatic moments which are not amenable to atomic partitioning schemes. Appreciable improvements can be observed through inclusion of four-body terms (especially for dipole moments) for these properties in cMBDF. This is further tested by employing the global form of cMBDF [cMBDF (global) in Fig. 4*B*] alongside the XGBoost (35) ML model which does not perform atomic partitioning. The XGBoost learning curves in Fig. 4*B* also contain the other two global representations used alongside KRR in Fig. 4*A*, namely CM and SLATM. The cMBDF (global) representation provides the most accurate XGBoost models across three out of the four properties. For HOMO–LUMO gaps as well, it is very similar in performance to the most accurate SLATM-based model, a global representation which induces >10× larger feature vector mappings than cMBDF (Fig. 2) on average. Nevertheless, the local KRR-based models employing atomic partitioning remain the most accurate throughout with appreciable improvements being observed upon the inclusion of four-body functionals (especially for dipole moments) in cMBDF.

However, we note here that while cMBDF performs well in comparison to other commonly used representations, the overall errors achieved by all models are relatively large compared to those for extensive properties such as atomization energies. For instance, a MAE of ∼0.2 eV for HOMO–LUMO gaps corresponds to a relative error of ∼5% for typical small organic molecules. Similarly, a 0.5 D MAE for dipole moments corresponds to a nearly 27% relative error for water (40). As noted earlier, regression of intensive properties such as these is highly nontrivial especially since atomic ML-models do not provide an obvious advantage for global molecular properties. This has been noted in other work with various methods proposed to deal with the learning of HOMO–LUMO gaps (41–43). Similarly, strategies such as the inclusion of response terms in the loss function (44) have been developed for dealing with dipole moments. Importantly, all of these schemes can be combined with cMBDF-based ML-models to tackle these challenging learning tasks. Due to this versatility and computational efficiency, cMBDF has been successfully applied to adaptive-ML-based methods for learning optimal exact-exchange mixing fractions with the PBE0 (45) functional (11) and optimal scaling factors for Pople-type Gaussian basis sets (12). Learning curves for both tasks can be found in *SI Appendix* (*SI Appendix*, Figs. S1 and S2 and the corresponding studies) and show a similar trend.

**Fig. 5.**
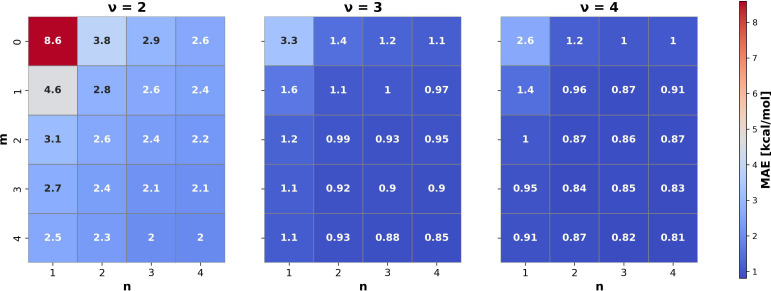
Heat-maps showing variation in predictive accuracy with the three integers ν (many-body order), m (derivative order), n (weighting function order) controlling the functionals generated by cMBDF. Mean absolute errors (MAE) for prediction of atomization energies using a KRR model are shown from the QM7b (4) dataset. A training/testing-split size of 2,000/1,000 was used.

Fig. 5 shows the predictive performance variation of cMBDF with the three integers ν, m and n. As expected, the many-body order ν has the largest effect on the accuracy. However, a similar performance as the four-body representation can be reached by raising m and n to larger values while keeping ν=3. This would be beneficial for larger systems where four-body and higher-order functionals can become expensive to evaluate. Nevertheless, four-body terms are known to be crucial in some cases (48) and increase the representation sensitivity (49). The four-body terms can also be important for some physical properties other than energetics as discussed in the previous section.

**Fig. 6.**
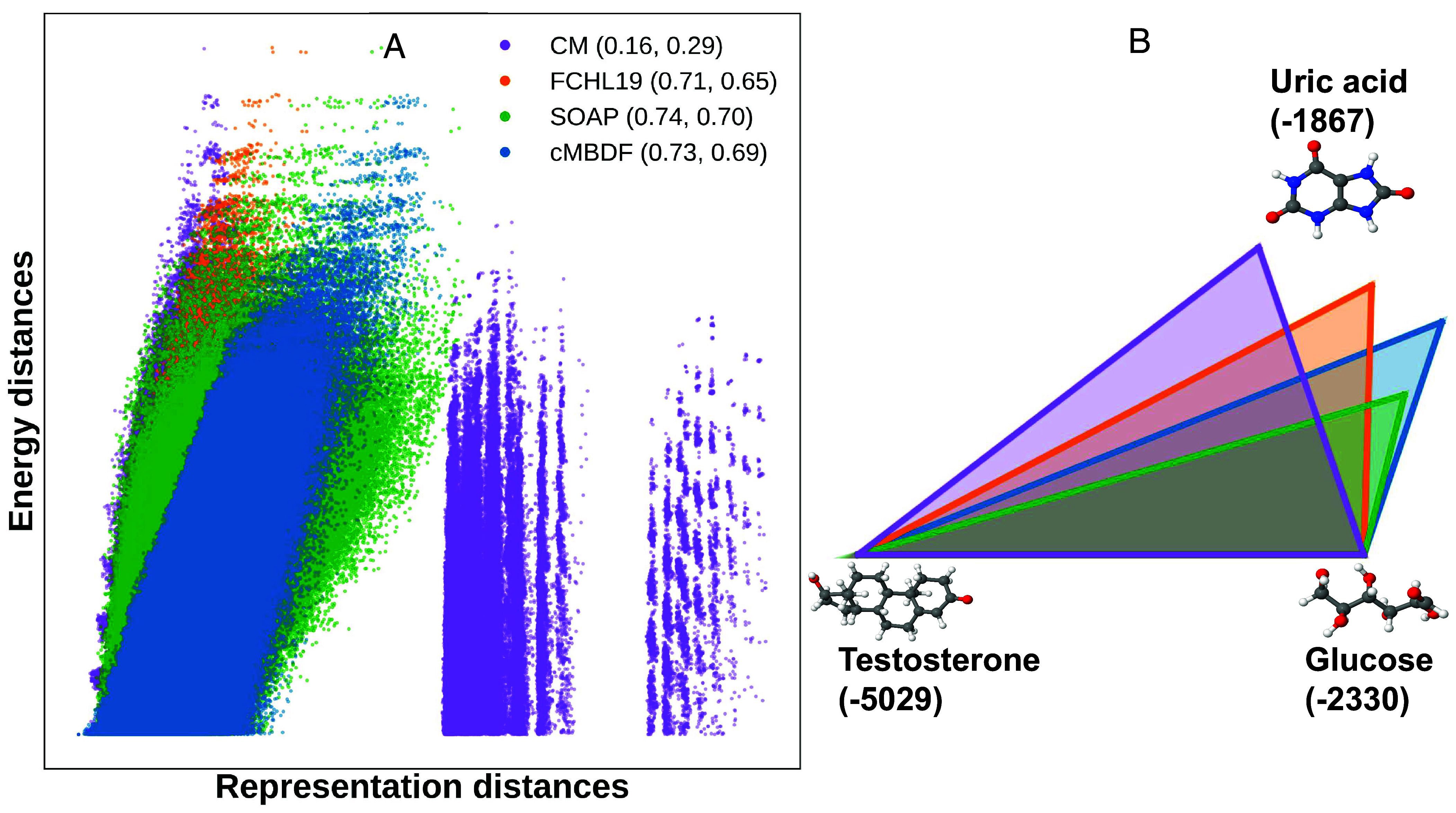
Correlation analysis between energetic and representation distances. (*A*) Correlation plot between atomization energy and molecular representation distances (Frobenius norm) for all molecules from the QM7b (4) dataset. All differences were normalized by the mean distance over the dataset. Numbers in the legend denote the Pearson and Spearman’s rank correlation coefficient values, respectively. (*B*) Molecular representation matrix (relative) distances (Frobenius norm) between three biologically relevant molecules. Sides of the triangles are proportional to the representation distance between the two molecules at the vertices. Distances shown are ratios to the Testosterone–Glucose distance for all four representations. Numbers in brackets below molecule name denote PBE0/def2-QZVP (45, 46) calculated atomization energies [using PySCF (47)] in kcal/mol.

To analyze the effectiveness of cMBDF, we examine the relation of representation and energetic differences between various molecules. Fig. 6*A* shows a correlation plot between atomization energy and representation matrix distances between all pairs of molecules from the QM7b (4) dataset. For comparison, we also plot 3 other commonly used representations across chemical compound space. To measure the linear and nonlinear correlations, we calculated the Pearson and Spearman’s rank correlation coefficient values for the four representations tested. A good linear correlation between the energetic and representation distances can be observed for the FCHL19, SOAP, and cMBDF molecular representations. This is important since kernel-based methods operate on the hypothesis that similar systems are expected to have similar labels. The correlation, however, can be nonlinear due to the mapping induced by the kernel function. The Spearman’s rank correlation coefficient takes this into account as it measures the degree of monotonicity between the two variables. For both coefficients, cMBDF shows strong correlations similar to the SOAP representation which induces a feature vector mapping 2 orders of magnitude larger than cMBDF (4,662 vs. 40 dimensions). This likely underpins the greater accuracy achieved by cMBDF-based kernel models in the low training data regime (Fig. 2*B*).

### Sensitivity Analysis.

This correlation can also be seen for larger systems across chemical compound space. Fig. 6*B* shows representation distance between three biologically relevant molecules of different size and composition. Sides of the triangles in the figure are proportional to the relative distances between representation matrices of the molecules at the vertices. Evidently, the relative distances between molecular representations induced by cMBDF align well with the DFT-calculated energetic differences of the three molecules. The testosterone–uric acid relative distance is the largest with cMBDF between the four representations even with cMBDF being significantly more compact than the other three representations. The smaller uric acid–glucose distance with cMBDF also aligns well with the relative energetic difference which is the smallest among the three pairs. This suggests that, despite their compact size, cMBDF feature vectors efficiently capture rich structural and compositional information, resulting in its strong performance on learning tasks.

The analysis presented above is akin to the correlation analysis between molecular fingerprints and physical properties conducted in ref. 49. Additional investigations, such as the sensitivity matrices introduced in that work and the exploration of “difficult” cases identified therein, will be pursued in a subsequent work.

## Discussion

In this work, we have introduced a systematically improvable class of compact atomic representations for use throughout chemical compound space. cMBDF encode the chemical environment of an atom through a set of translationally and rotationally invariant functionals of the atomic density. The functional values are efficiently evaluated via fast Fourier transforms using the convolution theorem which can be evaluated and stored on a predefined grid. Weighting functions of various types can be incorporated to capture physical interactions (short and long range) of interest efficiently via the atomic density functionals. The atomic feature vector size is invariant to the cut-offs employed, system size, and composition leading to a compact and constant size atomic descriptor. cMBDF is shown to outperform other commonly used representations for the learning of a variety of physical properties across chemical space while remaining nearly two orders of magnitude more compact. Due to its computational efficiency and versatility, cMBDF can lead to significantly faster prototyping, training, and testing of quantum machine learning models for a variety of tasks across chemical compound space. Future work will include a study of cMBDF gradients and their applicability across geometrical changes for relaxing geometries, identifying transition states (50) and molecular dynamics.

## Data and Code

Python implementation for generating cMBDF representations along with gradients is openly available at https://github.com/dkhan42/cMBDF.

It relies on the Numpy (51), Scipy (52) and Numba (53) Python libraries.
Dscribe (54, 55) library was used to generate the ACSF, LMBTR, SOAP representations with default parameters.
QMLcode (56) library was used to generate the CM, SLATM, FCHL19 representations with default parameters.
MORDRED was generated using the corresponding Python library (37).
All KRR-based ML models were trained, optimized, and deployed through the QMLwrap (23) (https://github.com/dkhan42/QMLwrap)-based implementation of the QMLcode library.
For details regarding the building and training of ML models (cross-validation, hyper-parameter and training/test set selection) the reader is referred to *SI Appendix*, *Kernel Based Methods*.

## Supplementary Material

Appendix 01 (PDF)

## Data Availability

Python implementation for generating cMBDF representations along with gradients’ data have been deposited in GitHub (https://github.com/dkhan42/cMBDF) (57). All other data are included in the manuscript and/or *SI Appendix*.
