# Supplementary material for: Generalized convolutional many-body distribution functional representations
Source: Proc Natl Acad Sci U S A. 2025 Oct 6;122(41):e2415662122. doi: 10.1073/pnas.2415662122 (PMC12541311; doi:10.1073/pnas.2415662122)
Supplement: Supplementary file 1 — Appendix 01 (PDF) [file pnas.2415662122.sapp.pdf]

Appendix: Generalized convolutional many body distribution functional representations

HERMITE POLYNOMIALS

The Hermite polynomials, H_m, are defined through the recursive Rodrigues' formula [1]

$$H_m(t) = (-1)^m e^{t^2} \partial_t^m \exp(-t^2) \quad (1)$$

This Rodrigues' formula implies a family of orthogonal polynomials with respect to the weight $w(t) = \exp(-t^2)$

$$\int_{-\infty}^{\infty} dt\ H_m(t) H_n(t) w(t) = \delta_{mn} \quad (2)$$

where δ_{mn} denotes the usual Kronecker delta. Hence, the H_m constitute a complete basis set for the L^2 Hilbert space equipped with the usual inner product.
Re-arranging Eq. 1 provides a closed expression for the m-th derivative of a Gaussian

$$\partial_t^m \exp(-t^2) = (-1)^m \exp(-t^2) H_m(t) \quad (3)$$

Using the following substition

$$t = \frac{x-\mu}{\sqrt{2}\sigma} \implies \frac{\partial}{\partial x} = \frac{\partial t}{\partial x}\frac{\partial}{\partial t} = \frac{1}{\sqrt{2}\sigma}\frac{\partial}{\partial t} \quad (4)$$

in eq. 3 we get

$$\partial_x^m \exp(-\frac{(x-\mu)^2}{2\sigma^2}) = \frac{(-1)^m}{(\sqrt{2}\sigma)^m} \exp(-\frac{(x-\mu)^2}{2\sigma^2}) H_m(\frac{x-\mu}{\sqrt{2}\sigma}) \quad (5)$$

Hence, for the normal probability density function $\mathcal{N}_x(\mu, \sigma) = \frac{1}{\sqrt{2\pi\sigma^2}} \exp(-\frac{(x-\mu)^2}{2\sigma^2})$ we have

$$\partial_x^m \mathcal{N}_x(\mu,\sigma) = \frac{(-1)^m}{(\sqrt{2}\sigma)^m} \mathcal{N}_x(\mu,\sigma) H_m(\frac{x-\mu}{\sqrt{2}\sigma}) \quad (6)$$

Substituting $x = r, \mu = R_{ij}$ in the above equation provides the relation used in Eq. 7 of main text.

KERNEL BASED METHODS

Unless specified otherwise, the ML model used throughout this work with all representations including cMBDF is the established Kernel Ridge Regression [11] (KRR), as also widely adopted by authors in the Lecture Notes in Physics book on quantum machine learning [12]. The reason for this choice is primarily its excellent performance in the low training data regime [13, 14] and ease of usage. Briefly, in KRR the prediction y_q for a query system is obtained as a weighted sum of similarity measures to all atoms/molecules in the training set

$$y_q = \sum_J^{N_{\text{train}}} \alpha_J k(\mathbf{X}_q, \mathbf{X}_J) \quad (7)$$

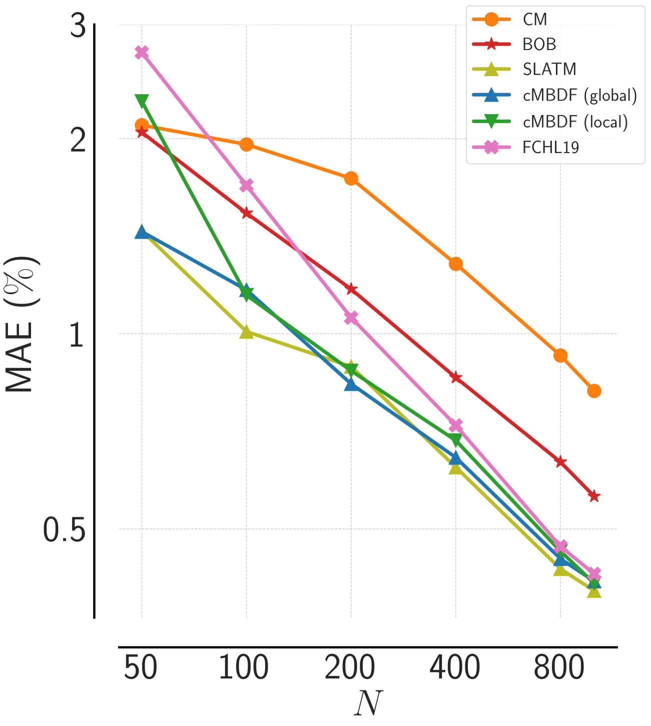

FIG. S1. Learning curves showing prediction error for optimal HF admixture ratio (a_{opt}) with the PBE0 functional [2] as a function of training set size for the representations Coulomb Matrix (CM)[3], Bag of Bonds (BOB)[4], Spectrum of London and Axilrod-Teller-Muto potentials (SLATM)[5], Faber-Christensen-Huang-Lilienfeld 19 (FCHL19)[6] and convolutional Many Body Distribution Functionals (cMBDF)[7]. Training and testing (200 out-of-sample amons) is performed on QM5 dataset [8]. a_{opt} values were calculated by optimizing the aPBE0 atomization energy to CCSD(T) atomization energy for each system. Figure taken from ref. [9].

where α_j are the regression weights, $\mathbf{X}$ are molecular representation matrices or atomic representation vectors, and $k(.,.)$ denotes a kernel function acting as a similarity measure. The regression weights α are obtained from the set of training labels $\mathbf{y}^{\text{train}}$ via the following equation

$$\alpha = (\mathbf{K} + \lambda \cdot \mathbf{I})^{-1} \mathbf{y}^{\text{train}} \quad (8)$$

where $\mathbf{K}$ is the kernel matrix of the training set, λ is a regularization parameter and $\mathbf{I}$ is the identity matrix. The kernel function primarily used in our work is the screened atomic Gaussian kernel

$$k(\mathbf{X}_I, \mathbf{X}_J) = \sum_{\mu \epsilon I} \sum_{\nu \epsilon J} \delta_{Z_\mu, Z_\nu} \exp\left(-\frac{||\mathbf{X}_{I\mu} - \mathbf{X}_{J\nu}||_2^2}{2l^2}\right) \quad (9)$$

where $\mathbf{X}_{I\mu}$ denotes the representation vector of atom μ within molecule I, l denotes the length-scale hyper-

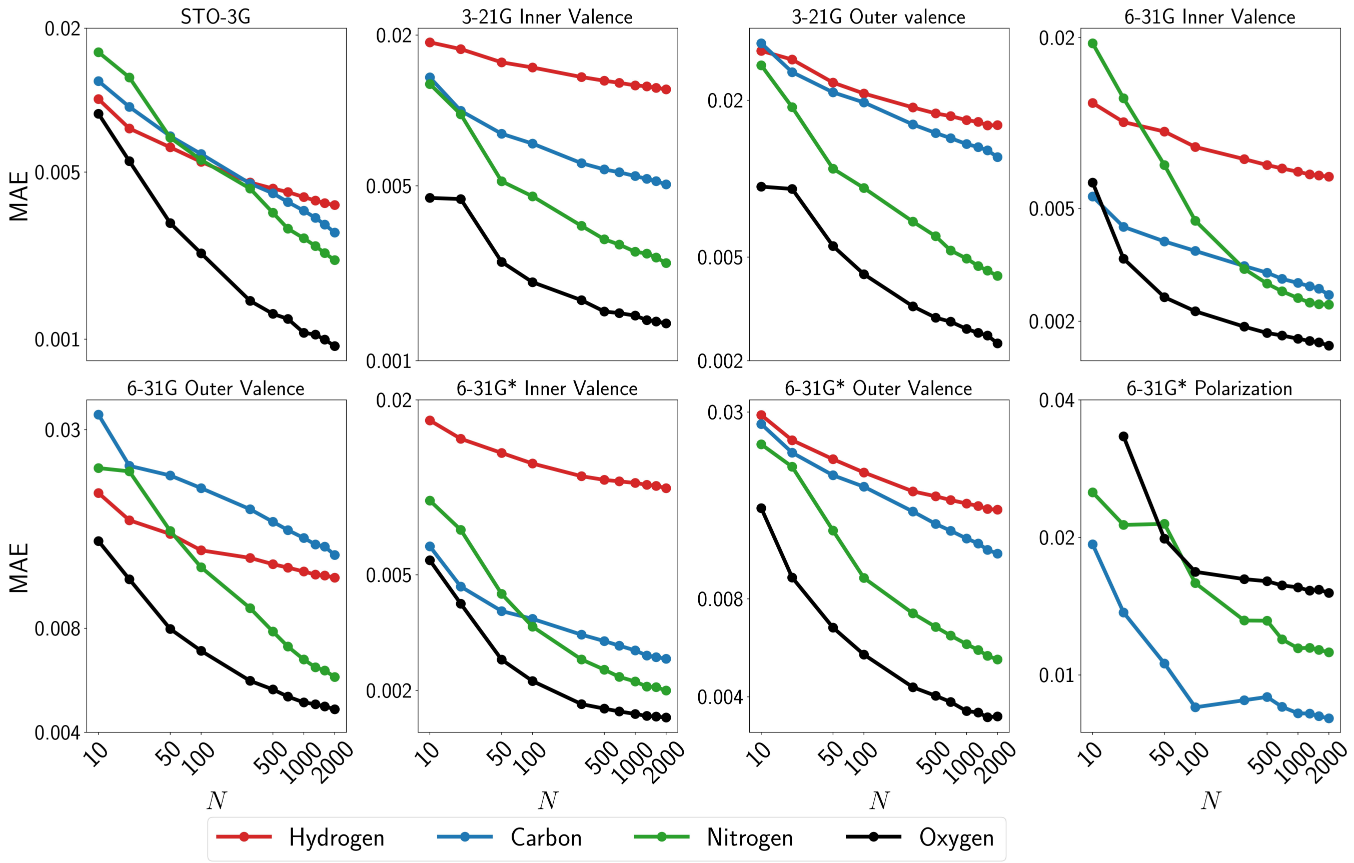

FIG. S2. Mean absolute errors (MAE) of predicted optimal scaling factors for STO-3G (valence orbitals), 3-21G, 6-31G (both inner and outer valence) and 6-31G* (inner and outer valence and polarization functions) as a function of training set size (number of molecules) using cMBDF on a validation set of 500 out-of-sample QM9 molecules. Figure taken from ref. [10].

parameter of the kernel and δ_{Z_μ,Z_ν} denotes a Kronecker Delta over the nuclear charges Z_μ, Z_ν which restricts the similarity measurement between atoms of the same chemical element[6]. Local representations of molecules correspond to matrices ($\mathbf{X}_I$ for molecule I) consisting of atomic feature vectors as their rows ($\mathbf{X}_{I\mu}$ for atom μ). Hence, cMBDF effectively only generates the feature vectors $\mathbf{X}_{I\mu}$ for all atoms within molecule I which are collected to form the matrix $\mathbf{X}_I$. Atom-index invariance is ensured by the structure of the kernel function in Eq. (9), which computes the sum of kernel measures over all pairs of atoms between molecules I and J. Consequently, ordering of the rows (atoms) within the molecular representation matrix is irrelevant.

Alternatively, feature vector mappings describing the entire system can be used resulting in molecular (or global) kernels of the form

$$k(\mathbf{X}_I, \mathbf{X}_J) = \exp\left(-\frac{||\mathbf{X}_I - \mathbf{X}_J||_2^2}{2l^2}\right) \quad (10)$$

where $\mathbf{X}_I$ now denotes a feature vector mapping of the molecule I. This form is usually more amenable to the learning of intensive properties that cannot be effectively partitioned onto atomic contributions [7, 15].

To achieve atom-index invariance in the global representation form $\mathbf{X}_I$ with cMBDF, we employ "bagging" in a similar fashion as the bag-of-bonds (BOB) [4] representation. Each set of functionals $\mathbf{P}[i]$ describing atom i is first sorted, followed by arrangement in a pre-specified order based on the chemical identities of the atoms in the molecule. This restricts the distance measurement in eq. 10 to atomic species of the same type analogous to the Kronecker Delta in eq. 9. While this method leads to siginificant gain in accuracy compared to a simple sorting [4], it introduces a scaling of the cMBDF molecular vector $\mathbf{X}_I$ dependent on the unique chemical species within the dataset.

Unless specified otherwise, global KRR (eq. (10)) was used alongside the global (molecular) version of the following representations throughout our results : CM [16], SLATM [15], MORDRED [17] (as obtained from the corresponding libraries mentioned in the "Data and code" section from the main text which incorporate atom-index invariance). Local (atomic) KRR (eq. (9)) was used alongside all other representations in their local form.

The length-scale (l) kernel hyper-parameter in eqs. 9, 10 and the regularizer (λ) in eq. 8 were optimized via grid-search. For the local kernels we used logarithmic

grids of $[0.1(2^n)\ \forall n\epsilon\{0,14\}]$ for l and $\left[10^{-3n}\ \forall n\epsilon\{1,4\}\right]$ for λ. For global kernels we use the grid $[10^n\ \forall n\epsilon\{2,8\}]$ for l. Implementation of the KRR models alongside hyper-parameter optimization was done through the `QMLwrap` [7] (`https://github.com/dkhan42/QMLwrap`) implementation of the `QMLcode` [18] library.

For all learning curves presented throughout the main text, training sets were chosen at random (from the entire dataset excluding the fixed test set) for each training set size. A fixed test set was always employed (i.e. irrespective of the training set size) on which the prediction errors were measured. This process was repeated 5-fold after which the mean of the 5 runs was plotted in all learning curves reported by us. This is the default procedure implemented in the `QMLwrap` [7] code which can be conveniently used for such benchmarking. A similar procedure was followed for obtaining the data-points in Figure 3 of the main-text where a fixed test set of 100k randomly chosen molecules from the QM9 dataset was used for all representations reported by us (i.e. except WK [19] and MACE [20]). Subsequently, learning curves were generated for all representations using the procedure described above (and similar to figure 2A) and the smallest training size required was estimated by the interception of the learning curve with the y = 1 kcal/mol line. For BOB [4] and CM [16], this intercept was estimated by extrapolating the corresponding learning curves. Further details can be obtained from Ref. [7].

XGBOOST

The following model configuration, taken from Ref. [21] for the SLATM/XGBR model, employing the `xgboost` [22] `Python` library was used in our work:

```python
from xgboost import XGBRegressor

model = XGBRegressor(objective = "reg:
    squarederror",
eval_metric = "rmse",
tree_method = "gpu_hist",
booster = "gbtree",
random_state = 42,
seed = 27,
subsample = 1.0,
scale_pos_weight = 1,
gamma = 0.0463639865776333,
reg_alpha = 0.0,
reg_lambda = 0.3375912633135869,
colsample_bytree = 0.5960970964006453,
min_child_weight = 1,
n_estimators = 2000,
max_depth = 10,
learning_rate = 0.01)
```


[1] W. Gautschi, *Orthogonal polynomials: computation and approximation* (OUP Oxford, 2004).

[2] C. Adamo and V. Barone, J. Chem. Phys. **110**, 6158 (1999).

[3] M. Rupp, A. Tkatchenko, K.-R. Müller, and O. A. von Lilienfeld, Phys. Rev. Lett. **108**, 058301 (2012).

[4] K. Hansen, F. Biegler, R. Ramakrishnan, W. Pronobis, O. A. von Lilienfeld, K.-R. Müller, and A. Tkatchenko, The Journal of Physical Chemistry Letters **6**, 2326 (2015), pMID: 26113956, https://doi.org/10.1021/acs.jpclett.5b00831.

[5] B. Huang and O. A. von Lilienfeld, Nature chemistry **12**, 945 (2020).

[6] A. S. Christensen, L. A. Bratholm, F. A. Faber, and O. Anatole von Lilienfeld, The Journal of Chemical Physics **152**, 044107 (2020), https://doi.org/10.1063/1.5126701.

[7] D. Khan, S. Heinen, and O. A. von Lilienfeld, The Journal of Chemical Physics **159** (2023).

[8] B. Huang, O. A. von Lilienfeld, J. T. Krogel, and A. Benali, Journal of Chemical Theory and Computation **19**, 1711 (2023).

[9] D. Khan, A. J. A. Price, M. L. Ach, and O. A. von Lilienfeld, "Adaptive hybrid density functionals," (2024), arXiv:2402.14793 [physics.chem-ph].

[10] D. Khan, M. L. Ach, and O. A. von Lilienfeld, arXiv preprint arXiv:2404.16942 (2024).

[11] V. Vapnik, *The nature of statistical learning theory* (Springer science & business media, 1999).

[12] K. T. Schütt, S. Chmiela, O. A. Von Lilienfeld, A. Tkatchenko, K. Tsuda, and K.-R. Müller, Lecture Notes in Physics (2020).

[13] F. A. Faber, L. Hutchison, B. Huang, J. Gilmer, S. S. Schoenholz, G. E. Dahl, O. Vinyals, S. Kearnes, P. F. Riley, and O. A. von Lilienfeld, Journal of Chemical Theory and Computation **13**, 5255 (2017), pMID: 28926232, https://doi.org/10.1021/acs.jctc.7b00577.

[14] G. Tom, R. J. Hickman, A. Zinzuwadia, A. Mohajeri, B. Sanchez-Lengeling, and A. Aspuru-Guzik, Digital Discovery **2**, 759 (2023).

[15] B. Huang and O. A. von Lilienfeld, Nature Chemistry **12**, 945 (2020).

[16] M. Rupp, A. Tkatchenko, K.-R. Müller, and O. A. von Lilienfeld, Phys. Rev. Lett. **108**, 058301 (2012).

[17] H. Moriwaki, Y.-S. Tian, N. Kawashita, and T. Takagi, Journal of Cheminformatics **10** (2018), 10.1186/s13321-018-0258-y.

[18] A. S. Christensen, F. A. Faber, B. Huang, L. A. Bratholm, A. Tkatchenko, K.-R. Müller, and O. A. von Lilienfeld, "Qml: A python toolkit for quantum machine learning," (2017).

[19] F. Bigi, S. N. Pozdnyakov, and M. Ceriotti, The Journal of Chemical Physics **161** (2024).

[20] I. Batatia, D. P. Kovacs, G. Simm, C. Ortner, and G. Csanyi, in *Advances in Neural Information Processing Systems*, Vol. 35, edited by S. Koyejo, S. Mohamed, A. Agarwal, D. Belgrave, K. Cho, and A. Oh (Curran Associates, Inc., 2022) pp. 11423–11436.

[21] S. Lee, S. Heinen, D. Khan, and O. A. von Lilienfeld, Machine Learning: Science and Technology **5**, 015052 (2024).

[22] T. Chen and C. Guestrin, in *Proceedings of the 22nd ACM SIGKDD International Conference on Knowledge Discovery and Data Mining*, KDD '16 (Association for Computing Machinery, New York, NY, USA, 2016) p. 785–794.
